# The PTP1B Inhibitor Trodusquemine (MSI-1436) Improves Glucose Uptake in Equine Metabolic Syndrome Affected Liver through Anti-Inflammatory and Antifibrotic Activity

**DOI:** 10.1155/2023/3803056

**Published:** 2023-09-30

**Authors:** Lynda Bourebaba, Anna Serwotka-Suszczak, Nabila Bourebaba, Magdalena Zyzak, Krzysztof Marycz

**Affiliations:** ^1^Department of Experimental Biology, Faculty of Biology and Animal Science, Wrocław University of Environmental and Life Sciences, Norwida 27B, Wrocław 50-375, Poland; ^2^Department of Veterinary Medicine and Epidemiology, Veterinary Institute for Regenerative Cures, School of Veterinary Medicine, University of California, Davis, CA 95516, USA

## Abstract

**Background:**

Hyperactivation of protein tyrosine phosphatase (PTP1B) has been associated with several metabolic malfunctions ranging from insulin resistance, metaflammation, lipotoxicity, and hyperglycaemia. Liver metabolism failure has been proposed as a core element in underlying endocrine disorders through persistent inflammation and highly fibrotic phenotype.

**Methods:**

In this study, the outcomes of PTP1B inhibition using trodusquemine (MSI-1436) on key equine metabolic syndrome (EMS)-related alterations including inflammation, fibrosis, and glucose uptake have been analyzed in liver explants collected from EMS-affected horses using various analytical techniques, namely, flow cytometry, RT-qPCR, and Western blot.

**Results:**

PTP1B inhibition using trodusquemine resulted in decreased proinflammatory cytokines (IL-1*β*, TNF-*α*, and IL-6) release from liver and PBMC affected by EMS and regulated expression of major proinflammatory microRNAs such as miR-802 and miR-211. Moreover, MSI-1436 enhanced the anti-inflammatory profile of livers by elevating the expression of IL-10 and IL-4 and activating CD4^+^CD25^+^Foxp3^+^ regulatory T cells in treated PBMC. Similarly, the inhibitor attenuated fibrogenic pathways in the liver by downregulating TGF-*β*/NOX1/4 axis and associated MMP-2/9 overactivation. Interestingly, PTP1B inhibition ameliorated the expression of TIMP-1 and Smad7, both important antifibrotic mediators. Furthermore, application of MSI-1436 was found to augment the abundance of glycosylated Glut-2, which subsequently expanded the glucose absorption in the EMS liver, probably due to an enhanced Glut-2 stability and half-life onto the plasma cell membranes.

**Conclusion:**

Taken together, the presented data suggest that the PTP1B inhibition strategy and the use of its specific inhibitor MSI-1436 represents a promising option for the improvement of liver tissue integrity and homeostasis in the course of EMS and adds more insights for ongoing clinical trials for human MetS management.

## 1. Introduction

Equine metabolic syndrome (EMS) is an increasingly recognized condition that affects various equines worldwide. It is estimated that in Europe almost half and in the USA even more than 51% of horses suffer from EMS [1, 2]. As in the case of human type 2 diabetes, an unsuitable lifestyle, characterized by a lack of physical activity and an inadequate diet, especially resulting in overfeeding with carbohydrates, is responsible for the development of the disease [3]. Due to the fact that the molecular mechanisms responsible for the development of this disease are still not fully understood, it remains difficult to diagnose EMS in the early stages and to design effective treatment strategies based on modern targeted therapies.

Apart from its significant relevance in veterinary medicine practice, EMS condition has also been pointed as a comparable endocrinopathy to human metabolic syndrome (MetS). Indeed, reports from the American College of Veterinary Internal Medicine (ACVIM) and European College of Equine Internal Medicine (ECEIM) EMS consensus statements have presented the evidence of clinical, phenotypical, and molecular similarities between EMS and human MetS physiopathology. Common disease mechanisms include insulin dysregulation and resistance, abnormal adiposity, and metaflammation, which provide interesting prospects for the use of EMS as a valuable experimental model in translational human medicine [4–7].

From an etiological point of view, EMS is an endocrine disorder involving a number of clinical symptoms that lead primarily to the development of insulin resistance and, as a result, hyperinsulinemia, laminitis, hyperlipidaemia, and local and systemic inflammation. One of the key factors involved in the development of insulin resistance lies in the deterioration of lipid metabolism leading to the accumulation of lipids on the neck or base of the tail and lipotoxicity at the tissue level. Related obesity is often recognized in horses with EMS; however, it is not considered as a required marker for the diagnosis [1, 8].

An undisputable component of EMS corresponds to persistent inflammation occurring locally, in adipose tissue (AT) or liver, as well as systemically. Enhanced production of proinflammatory mediators including tumor necrosis factor *α* (TNF-*α*), interleukin-1 (IL-1), and interleukin-6 (IL-6) is often observed in AT of EMS horses. Along with the aforementioned cytokines, adipocytes also produce other molecules such as leptin (LEP) and C-reactive protein (CRP), which together with inflammation are responsible for inhibiting the expression of genes regulating the metabolism of fatty acids and glucose and decreased secretion of certain antihyperglycemic adipokines [9–12]. In addition, to the abundant proinflammatory adipokines described previously, AT also secretes some anti-inflammatory factors, as for instance IL-4 or IL-10 [13, 14]. Although exerting a pleiotropy of biological activities, interleukins 4 and 10 are essentially recognized for their immunosuppressive effects mediated through proinflammatory cytokine expression lowering and inducing type-switching of M1 to M2 macrophages under acute or prolonged inflammatory states [15, 16]. Loss of balance between the amount of pro and anti-inflammatory molecules can result in decreased insulin sensitivity which leads to the development of EMS [17].

One of the most prominent insulin resistance-associated outcomes lies in the profound deregulation of liver metabolism. Liver fibrosis is characterized by an excessive accumulation of extracellular matrix (ECM) that is not adequately balanced by degradation, mainly by matrix metalloproteinases. The net formed from collagen and other ECM proteins creates scar tissue, which impacts liver function and regeneration. During liver damage, injured hepatocytes release proinflammatory factors resulting in the recruitment and activation of immune cells, leading to hepatitis. Moreover, liver fibrosis contributes to the development of laminitis by altering the metabolism of dietary carbohydrates [18–20]. Therefore, a detailed analysis of the molecular processes underlying EMS in the liver can help in understanding the development of the disease and enable its reversion by pharmacologically targeting the organ's metabolism.

Insulin resistance progression is intimately connected to a defect in insulin receptor (INSR) tyrosine kinase activity. The receptor is a protein tyrosine kinase, whose mechanism of action is strictly dependent on its natural ligand binding insulin, resulting in autophosphorylation and phosphorylation of other proteins, what in turns triggers a cascade of intracellular signal pathways. Insulin signaling is negatively regulated by protein tyrosine proteases 1B (PTPs), that negatively regulates the insulin pathway, inter alia, through JAK2-STAT3 axis, which is dysfunctional in types 2 diabetes and obesity [10, 21]. Interestingly, PTP1B was also shown to play a major role in regulating inflammatory mechanisms, and among others, it was demonstrated to be engaged in modulating the inflammatory response in alcoholic liver injury and nonalcoholic fatty liver disease [22–24].

Pharmacological strategies aiming to improve the insulin sensitivity by enhancing INSR phosphorylation seem to be promising approaches in the therapy of obesity, insulin resistance, and EMS, especially as PTP1B appears to be a critical link between the maintenance of insulin sensitivity, inflammation (both systemic and hepatic) as well as liver-related conditions. Therefore, we decided to analyse the effect of PTP1B inhibition on insulin sensitivity defined by expression mediators involved in inflammatory and fibrogenic events. For this purpose, we used a low molecular weight compound, MSI-1436. This inhibitor of the protein tyrosine protease PTP1B, originally isolated from the liver of the dogfish shark, was previously shown to improve glucose tolerance and insulin sensitivity in insulin-resistant mice [24–28]. Recently, we confirmed *in vitro*, that MSI-1436 improves EMS adipose derived progenitor stem cells in the course of adipogenic differentiation through modulation of ER stress, apoptosis, and oxidative stress [26]. Consequently, the goal of this research was to analyse the MSI-1436-mediated PTP1B inhibition and its influence on inflammatory mediators' levels and fibrosis status in the liver, as well as glucose absorption.

## 2. Materials and Methods

### 2.1. Liver Tissue Explants' Collection and *Ex-Vivo* Treatment

Liver tissue samples were collected postmortem from healthy and EMS-affected Polish cold-blooded horses from a local slaughterhouse (Targowa, Rawicz, Poland). Animals that were euthanized for reasons unrelated to this study were aged between 8 and 10 years, their qualification was made by experienced veterinarians based on their clinical profiles and medical history following the previously described procedure and established criterions [29], that included body weight, body condition score (BCS), cresty neck score (NCS), fasting insulin and leptin levels, and combined glucose-insulin test (CGIT). All enrolled animals were additionally verified for the existence of any comorbidity or anterior condition that might influence the experiment.

Collected tissue explants were washed with Hank's balanced salt solution (HBSS) supplemented with 1% of penicillin/streptomycin solution (P/S) and immediately placed in the transport medium containing Dulbecco's Modified Eagle's Medium (DMEM) with 1 g/L glucose, supplemented with a 1% PS. Forthwith, the materials were transported to the laboratory and processed for further analysis. Healthy and EMS liver biopsies were first diced into small pieces (<0.5 cm), washed three times with PBS containing 1% P/S solution, and placed into 6-well plates containing 2 mL of Dulbecco's Modified Eagle's Medium (DMEM) containing 1 g/L glucose and 1% P/S solution alone (For control groups) or supplemented with a final concentration of 1 *µ*M MSI-1436 compound (For treated groups) per well. All the experimental groups underwent an incubation of 24 h at 37°C with 5% CO_2_ and 95% of humidity. All tissue samples were then washed with fresh HBSS and secured in TRIzol for mRNA isolation and in RIPA buffer supplemented with 1% proteinases inhibitor cocktail for protein isolation. Additional groups of tissue were exposed to 25 mM D-glucose for the glucose uptake assay. Following 24 h incubation in the presence of each related treatment and D-glucose, the amount of consumed glucose was determined using the glucose colorimetric assay kit (Cayman Chemicals, Michigan, USA) following manufacturer's instructions.

### 2.2. Peripheral Blood Mononuclear Cells' (PBMC) Isolation and Treatment

Samples of fresh blood were first collected from healthy and EMS-affected horses in syringes filled with heparin. Then, an isolation of PBMC cells was performed as described by Kornicka et al. [30]. In brief, the cells were subjected to a Ficoll Histopaque®-1077 density gradient centrifugation during 30 min, at 400 × *g*, 25°C. Then, the cells localized in the buffy coat layer were collected and washed three times with HBSS. Finally, the cells were seeded in 6-well plates supplemented with 2 mL of the complete culture medium (RPMI 1640 medium + 10% fetal bovine serum (FBS) + 1% P/S). PBMCs were subsequently subjected to the treatment mentioned in the previous section; the cells were supplemented with RPMI-1640 medium containing 1% P/S (for control groups) containing or not 1 *µ*M MSI-1436 compound (For treated groups). Treated and untreated cells were then collected, washed with fresh HBSS, and secured in either TRIzol for mRNA isolation or in RIPA buffer supplemented with 1% proteinases inhibitor cocktail for protein isolation.

### 2.3. Flow Cytometry Analysis of T Regulatory Cells

The three experimental groups of PBMC cells (EqPBMCs_HE_, EqPBMCs_EMS_, and EqPBMCs_EMS_ + 1 *µ*M MSI-1436) previously isolated from healthy and EMS-affected horses and treated with 1 *µ*M MSI-1436 were first stained against CD4, CD25, and Foxp3 markers. In brief, cells were incubated with mouse anti-horse CD4 conjugated with FITC (MCA1078F, 1 : 200; BioRad, Hercules, CA, USA), CD25 conjugated with AF700 (AF-223-NA, 1 : 200; Bio-Techne, Minneapolis, Minnesota, USA), and Foxp3 conjugated with PE (61-5773-82, eBioscience, Thermo Fisher Scientific, Carlsbad, CA, USA), for 30 min at 4°C. Then, the cells were washed three times and resuspended in phosphate buffered saline (PBS). Measurements were performed using the BD LSR Fortessa with FACSDiva version 9.0 flow cytometer equipped with FCS Express 7.0 software (Becton Dickinson, San Jose, USA). A minimum of 20,000 cells were analyzed, and CD4^+^/CD25^+^/Foxp3^+^ cell population has been determined following appropriate gating.

### 2.4. Determination of Interleukin-1*β* (IL-1*β*), Tumor Necrosis Factor-*α* (TNF-*α*), and Vascular Endothelial Growth Factor (VEGF) Levels

Levels of proinflammatory cytokines and VEGF were evaluated in healthy, EMS, and EMS + 1 *µ*M MSI-1436 liver tissues and PBMCs lysates using Horse Interleukin 1 Beta ELISA Kit (BT Lab, Cat. N° E0079Ho, Zhejiang Province, China), Equine TNF-*α* DuoSet ELISA (R&D Systems, Cat. N° DY1814, Minneapolis, MN, USA), and the Human VEGF Quantikine ELISA Kit (R&D Systems, Cat. N° DVE00, Minneapolis, MN, USA) according to the manufacturer's instructions. In brief, 50 *µ*L of each sample (liver and PBMC lysates) and provided standards at various concentrations were introduced into microtiter wells and mixed with each corresponding antibody. The plates were incubated for 60 minutes at 37°C. Then, all wells were washed 5 times with the 1x Wash Buffer. Substrates were afterwards added to each well and incubated for 10 minutes at 37°C in the dark. A stop solution was subsequently added, and absorbance values were measured using a microplate spectrophotometer (Epoch, BioTek) at 450 nm. Final concentrations were derived from each constructed standard curve, and data were analyzed using GrapPad Prism8.

### 2.5. Analysis of Relative Gene Expression

Gene expression levels of main transcripts involved in inflammation and fibrosis were assessed by means of Real-Time Quantitative Reverse Transcription PCR. Total RNA was extracted from homogenized tissue and cell samples. The liver tissue was subjected to two washes using PBS and subsequently treated with TRIzol reagent. The PBMCs cells were recovered in TRIzol from each culture. All samples were homogenized and incubated with chloroform for 3 min and subjected to a centrifugation for 15 min at 12 000 rpm in 4°C, the appearing aqueous phase was collected and RNA precipitated using ice-cold 100% isopropanol. The obtained pellet was washed twice with 70% ethanol and dissolved in DEPC-treated water. The concentration and quality of isolated RNAs were determined using a nanospectrophotometer (Epoch, BioTek). An amount of 150 ng of RNA was reverse transcribed with the Takara PrimeScriptTM RT Reagent Kit following the manufacturer' protocol. In addition, estimation of miRNA levels was assessed following the protocol previously described by Morawska-Kochman et al. [31]. In brief, after digestion of the mRNA, the cDNA was synthesized using 375 ng of total RNA using a Mir-X™ miRNA First-Strand synthesis kit (Takara Bio Europe, Saint Germain, Laye, France). qPCR reaction mixture consisted of SensiFAST SYBR Green (Bioline, London, UK), DEPC-treated water, a pair of selected primers (Tables 1 and 2), and cDNA. The Real-Time PCR was performed with a CFX ConnectTM Real-Time PCR Detection System (Bio-Rad) with the following cycling conditions: 95°C for 2 min, followed by 40 cycles at 95°C for 15 s, annealing for 15 s, and elongation at 72°C for 15 s. The relative expression level was calculated using the RQ_MAX _log2 scale algorithm.

### 2.6. Profiling of Proteins Expression Using Western Blot

Total proteins were isolated from liver tissue samples and PBMCs cells using ice-cold RIPA buffer supplemented with Proteases Inhibitors cocktail at a ratio of 1 : 1000, following sequential homogenization on ice. Insolubilized materials from both tissue and cells samples were removed by centrifugation at 6000*g* for 20 min at 4°C, and protein concentrations from remaining lysates were quantified using the Pierce™ BCA Protein Assay Kit (Life Technologies, USA). A final concentration of 20 *µ*g of proteins was used for each sample, previously denatured in a 4 × Laemmli loading buffer (Bio-Rad, USA) during 5 min at 95°C, subjected to SDS-PAGE electrophoresis, at 100 V for 90 min in Tris/glycine/SDS buffer for proteins separation, and transferred onto polyvinylidene difluoride (PVDF) membranes (Bio-Rad, USA) at 100 V, 250 mA for 1 h at 4°C in a Tris/glycine buffer/methanol. Then, the membranes were incubated in a solution of 5% nonfat milk during 1 h at room temperature and subsequently labelled with primary antibodies (Table 3) overnight at 4°C. Excess antibodies have been washed out using 1X TBST, and membranes were incubated for 1 h at room temperature with HRP-conjugated secondary antibody (dilution 1 : 1000 in TBST) and washed with 1xTBST. The chemiluminescent signals were evaluated with the ChemiDoc MP Imaging System (Bio-Rad, USA), and results were analyzed with the Image Lab Software (Bio-Rad, USA).

### 2.7. Statistical Analysis

The obtained results were analyzed by one way variance analysis (ANOVA) using GraphPad Software (Prism 8.20, San Diego, CA, USA), and differences between groups were determined using Tukey' post-hoc test. Statistically significant results were marked with an asterisk, respectively, for *p* < 0.05 (^*∗*^), *p* < 0.01 (^*∗∗*^), and *p* < 0.001 (^*∗∗∗*^). Results are presented as the statistical mean ± SD from at least three independent experiments.

## 3. Results

### 3.1. PTP1B Inhibition Decreases Proinflammatory Cytokines' Expression in EMS-Affected Livers and PBMC

**Figure 1:**
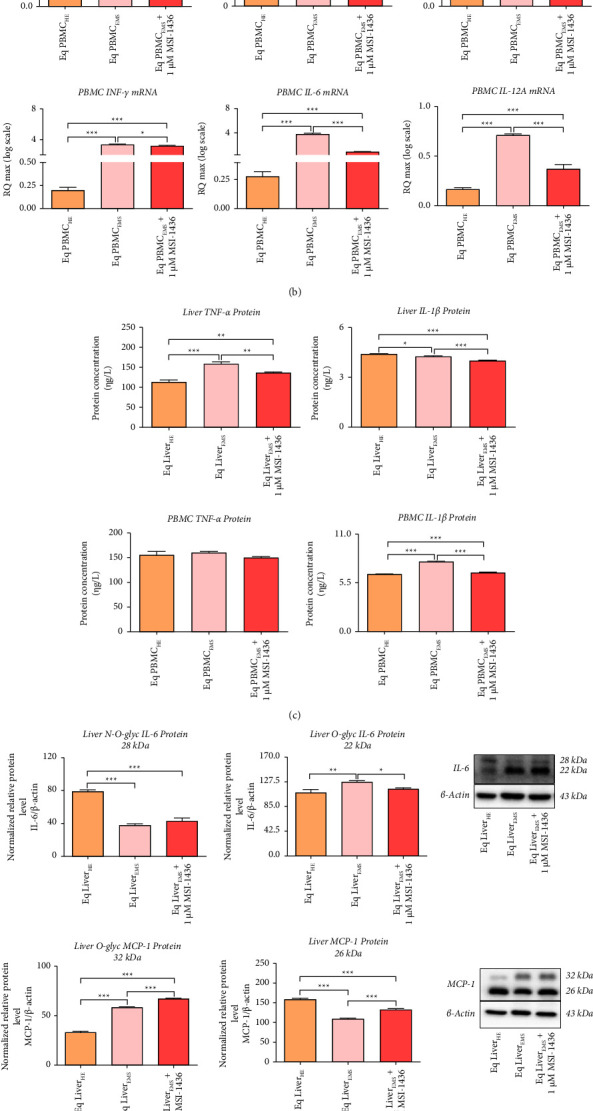
Inhibition of PTP1B with MSI-1436 alleviates inflammation in the liver and PBMC derived from EMS horses. (a) Relative gene expression of master mediators involved in inflammatory responses in liver tissue. (b) Relative gene expression of proinflammatory factors analyzed in equine PBMC. (c) Total levels of IL-1*β* and TNF-*β* proteins in the liver and PBMC lysates. (d) Relative protein expression of IL-6 and MCP-1 cytokines accompanied with their representative immunoblots for the liver and PBMC samples. Results were normalized to the expression of endogenous *β*-actin control. (e) Relative expression of key microRNA regulating proinflammatory responses determined in both liver and PBMC groups. Representative data from three independent experiments are shown ± SD (*n* = 3). An asterisk (^*∗*^) indicates a comparison among healthy, EMS, and EMS-treated groups. ^*∗*^*p* < 0.05, ^*∗∗*^*p* < 0.01, and ^*∗∗∗*^*p* < 0.001. Eq Liver_HE_: liver explants derived from healthy horses; Eq Liver_EMS_: untreated liver explants derived from EMS horses. Eq Liver_EMS_ + 1 *µ*M MSI-1436: liver explants derived from EMS horses and treated with 1 *µ*M of MSI-1436 inhibitor. Eq PBMCr_HE_: PBMC derived from healthy horses; Eq PBMC_EMS_: untreated PBMC derived from EMS horses. Eq PBMC_EMS_ + 1 *µ*M MSI-1436: PBMC derived from EMS horses and treated with 1 *µ*M of MSI-1436 inhibitor.

**Figure 2:**
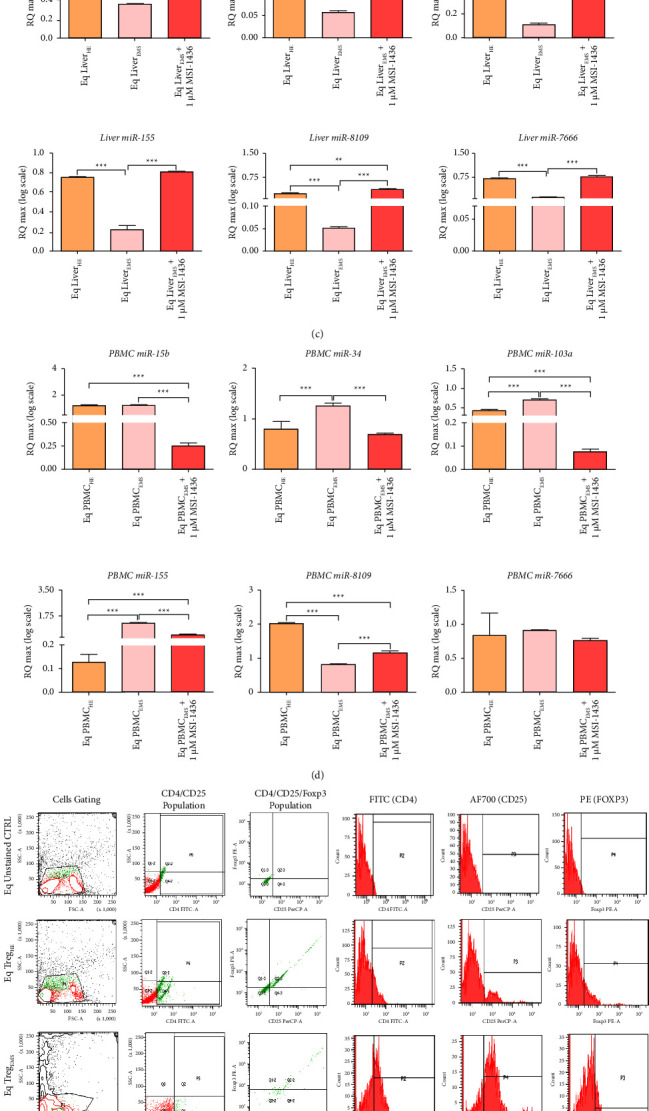
MSI-1436 ameliorates anti-inflammatory signals in livers and PBMC affected by EMS. (a) Relative liver IL-4 and IL-10 protein expression. Results were normalized to the expression of endogenous *β*-actin control. (b) Levels of IL-4 and IL-10 transcripts in liver and PBMC samples. Relative expression of anti-inflammatory microRNAs in (c) liver and (d) PBMC groups. (e) Gating strategy for PBMCs flow cytometric analysis of CD4^+^, CD25^+^ and Foxp3^+^ T cells. Signal representative of stained markers: FITC (CD4), AF700 (CD25), and PE (FOXP3). The population of CD4/CD25/Foxp3 cells appears in Q2 quadrant. (f) Bar charts depicting the total percentage of CD4^+^, CD25^+^, Foxp3^+^, and triple positive CD4^+^CD25^+^Foxp3^+^ cells. Representative data from three independent experiments are shown ± SD (*n* = 3). An asterisk (^*∗*^) indicates a comparison among healthy, EMS, and EMS-treated groups. ^*∗*^*p* < 0.05, ^*∗∗*^*p* < 0.01, and ^*∗∗∗*^*p* < 0.001. Eq Liver_HE_: liver explants derived from healthy horses; Eq Liver_EMS_: untreated liver explants derived from EMS horses. Eq Liver_EMS_ + 1 *µ*M MSI-1436: liver explants derived from EMS horses and treated with 1 *µ*M of MSI-1436 inhibitor. Eq PBMCr_HE_: PBMC derived from healthy horses; Eq PBMC_EMS_: untreated PBMC derived from EMS horses. Eq PBMC_EMS_ + 1 *µ*M MSI-1436: PBMC derived from EMS horses and treated with 1 *µ*M of MSI-1436 inhibitor.

Liver inflammation is considered as an integral causative and exacerbating component of equine metabolic syndrome, leading to liver chronic inflammation, insulin resistance, and overall critical metabolic failure. Increased proinflammatory cytokines at both tissue and systemic levels are one of the main EMS hallmarks. As shown in Figure 1(a), EMS livers are characterized by critical increased expression of IL-1*β*, IL-6, IL-12A, TNF-*α*, and MCP-1 genes when compared to healthy livers (*p* < 0.001). The same transcripts appeared to be substantially overexpressed in PBMC derived from the same horses, unlike MCP-1 gene, which was found to be downregulated in the same cells (Figure 1(b)). Moreover, under EMS condition, the CRP transcript was found to be upregulated, all of which suggesting the occurrence of severe systemic and tissue inflammation. Overexpression of proinflammatory cytokines was further observed at the protein level, where liver and PBMC levels of IL-1*β* and TNF-*α* proteins (Figure 1(c)) emerged significantly higher in EMS samples over healthy tissues (*p* < 0.001). Similarly, Western blot profiling evidenced an overexpression of IL-6 and MCP-1 proteins in liver tissue, evoking an elevation in tissue inflammatory cells activities such as macrophages or Kupffer cells (Figure 1(d)). Exposure of EMS livers to 1 *µ*M of PTP1B inhibitor resulted in a significant regulation of proinflammatory marker expression. In point of fact, MSI-1436 enabled to significantly reduce the relative expression levels of IL-1*β*, IL-6, IL-12A, and TNF-*α* transcripts in the EMS liver and levels of IL-1*β*, IL-6, IL-12A and IFN-*γ* in PBMC cells (*p* < 0.001; *p* < 0.05). Therewith, MSI-1436 was also successful in lowering relative IL-6 protein expression in treated liver tissue (Figure 1(d)). Interestingly, the PTP1B inhibitor augmented the MCP-1 levels, as shown by the increased relative expression of O-glycosylated MCP-1 chemokine over health (*p* < 0.001) and EMS (*p* < 0.05) tissues and dimerized MCP-1 protein compared to EMS liver (Figure 2(d)).

Insofar microRNAs are considered as critical mediators of the immune system regulatory network, the expression of selected proinflammatory microRNAs in the liver and PBMC samples was determined using RT-qPCR. Both the EMS liver and PBMC displayed increased relative expression of miR-122 and miR-802 (Figure 1(e)) when compared to control groups (*p* < 0.001). Interestingly, expression of miR-211 was found to be significantly upregulated in the EMS liver group (*p* < 0.001), while it was downregulated in PBMC derived from EMS horses (*p* < 0.001). Application of MSI-1436 exerted differential regulatory effects on the abovementioned microRNAs (Figure 1(e)). In fact, the PTP1B inhibitor treatment resulted in a significant downregulation of miR-802 in both liver (*p* < 0.01) and PBCM groups (*p* < 0.001) as opposed to EMS untreated groups, while expression of miR-122 was found to be upregulated as compared to healthy and EMS groups (Figure 1(e)). Similarly, expression of miR-211 remained unchanged in liver explants treated with MSI-1436 compared to both control (*p* < 0.001) and EMS groups, while its expression in PBMC was found to be increased as opposed to the EMS group (*p* < 0.001).

### 3.2. MSI-1436 Inhibitor Ameliorates Interleukin 10-Mediated Anti-Inflammatory Signaling in EMS-Derived Liver Tissue

Excessive proinflammatory responses over anti-inflammatory pathways are an integral component of peripheral tissue degeneration in the course of metabolic syndrome onset; therefore, the influence of PTP1B inhibition on EMS-mediated anti-inflammatory mechanisms depletion has been further evaluated. EMS-affected livers displayed collapsed IL-4 and IL-10 key anti-inflammatory cytokines expression at protein levels when compared to healthy livers (Figure 2(a)). Interestingly, gene expression of both cytokines was found to be significantly increased under EMS condition, evoking the establishment of a compensatory mechanism aiming at overcoming the decreased proteins levels (Figure 2(b)). Exposure of EMS liver explants to 1 *µ*M of trodusquemine (MSI-1436) substantially augmented the protein levels of IL-10, a potent anti-inflammatory mediator, while reducing its expression at the mRNA level compared to the untreated group (*p* < 0.001); remarkably, the expression of IL-10 protein appeared significantly higher than that of healthy tissue (*p* < 0.001), pointing out the ability of MSI-1436 to boost endogenous anti-inflammatory pathways. Inversely, the PTP1B inhibitor did not induce any changes in IL-4 expression at both protein and mRNA levels, that was found to be as dysregulated as untreated EMS tissue (Figures 2(a) and 2(b)). These observations have been further confirmed in EMS PBMC, where genic expressions of IL-10 and IL-4 were markedly upregulated under metabolic distress (Figure 2(b)) and subsequently reversed upon MSI-1436 supplementation for a period of 24 h (*p* < 0.001).

Anti-inflammatory potential of MSI-1436 has been additionally investigated in regard with its effect on microRNAs that negatively regulate inflammatory responses. Obtained data demonstrated that whereas EMS groups displayed critical loss in anti-inflammatory microRNA expression (miR-15b, miR-34, miR-155, miR-103, miR-7666, and miR-8109), MSI-1436-treated liver explants showed intensified relative expression of the abovementioned microRNAs (Figure 2(c)). By contrast, EMS PBMC showed dysregulated and increased anti-inflammatory microRNAs expression, which was abrogated following application of MSI-1436 (Figure 2(d)).

Insofar regulatory T cells (Tregs) represent the first endogenous line for inflammatory processes' regulation and generally for maintaining innate and adaptive immune networks' homeostasis, the aptitude of MSI-1436 to initiate Tregs phenotype has been further studied. As depicted in Figures 2(e) and 2(f), EMS-derived PBMC displayed critical depletion in CD4^+^CD25^+^Foxp3^+^ regulatory T cell population, indicating a loss in Treg-mediated suppressive effects on inflammatory monocytes/macrophages. MSI-1436 treatment substantially promoted the activation of CD4^+^CD25^+^Foxp3^+^ regulatory T cells (Figure 2(f)) when compared to EMS untreated cells (*p* < 0.001) and demonstrated comparable population percentage with healthy PBMC, which corroborated the strong anti-inflammatory and immunomodulatory potential of MSI-1436.

### 3.3. MSI-1436 Inflects Fibrotic Events in EMS-Affected Liver Tissue

**Figure 3:**
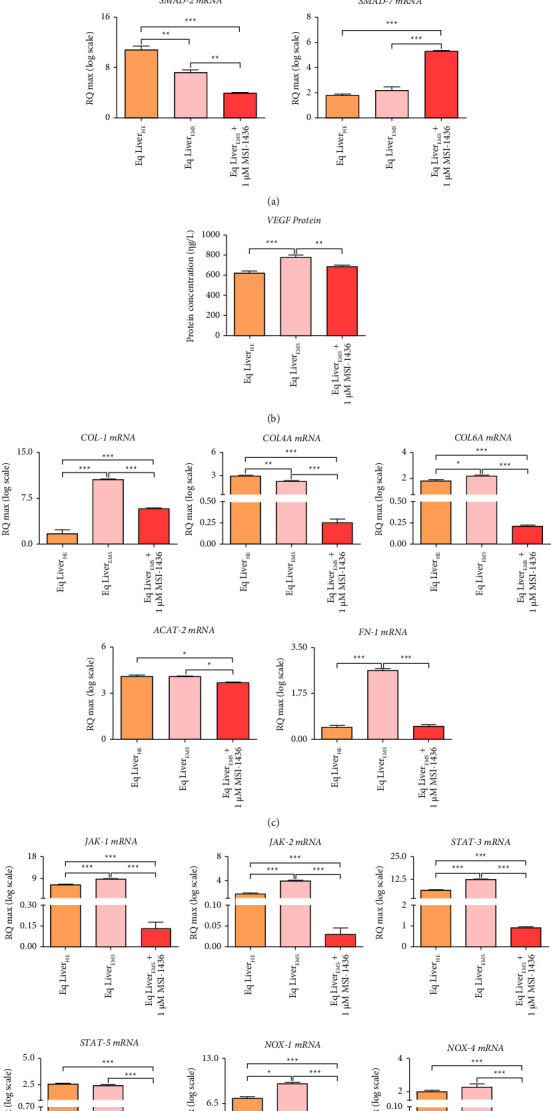
PTP1B inhibition attenuates liver fibrogenesis pathways. (a) Relative gene expression of major profibrogenic mediators. (b) Protein levels of tissue VEGF. (c) Relative levels of liver fibrosis-associated marker transcripts. (d) Fold changes in gene expression of PTP1B-related profibrogenic effectors. Representative data from three independent experiments are shown ± SD (*n* = 3). An asterisk (^*∗*^) indicates a comparison among healthy, EMS, and EMS-treated groups. ^*∗*^*p* < 0.05, ^*∗∗*^*p* < 0.01, and ^*∗∗∗*^*p* < 0.001. Eq Liver_HE_: liver explants derived from healthy horses; Eq Liver_EMS_: untreated liver explants derived from EMS horses. Eq Liver_EMS_ + 1 *µ*M MSI-1436: liver explants derived from EMS horses and treated with 1 *µ*M of MSI-1436 inhibitor.

Liver fibrosis is considered as a strong predictor for liver alterations prognosis associated with persistent lipotoxicity under metabolic syndrome. Hence, the potential of MSI-1436 to attenuate EMS-related fibrogenesis has been investigated. As demonstrated in Figure 3(a), EMS livers were characterized by strong activation of profibrogenic pathways. Indeed, obtained data evidenced a significant upregulation of TGF-*β*, a master activator of liver fibrosis and downstream effectors ACVR2A and VEGF, which promote hepatic stellate cell expansion and differentiation (*p* < 0.001), Therewith, increased protein levels of VEGF (Figure 3(b)) were similarly observed, confirming the initiation and progression of the fibrotic process in EMS livers. Consequently, EMS untreated groups displayed critical induction of liver-fibrosis tissue markers (Figure 3(c)), namely, COL-1, COL-6A, and FN-1 over nonfibrotic liver tissue of the healthy group (*p* < 0.001). Interestingly, ACAT-2 transcript levels remained unchanged, while those of COL-4A appeared downregulated in the same untreated EMS groups (Figure 3(c)). Application of MSI-1436 to EMS livers for a period of 24 h resulted in a potent attenuation of activated fibrotic pathways. MSI-1436 reduced the expression of both TGF-*β* and VEGF at mRNA and protein levels compared to the EMS untreated group (*p* < 0.001 and *p* < 0.01, respectively). Surprisingly, PTP1B inhibition further reduced the fold expression of TGF-*β* under basal levels as compared to healthy tissue, suggesting the strong anti-inflammatory and antifibrotic effect of the inhibitor (Figures 3(a) and 3(b)). In a similar fashion, MSI-1436 augmented the expression of SMAD-7, a potent TGF-*β*-mediated fibrosis inhibitor, while downregulating the SMAD-2 mRNA level, which is a key TGF-*β*-stimulated profibrotic transcription factor (Figure 3(a)). Correspondingly, relative levels of COL-1, COL-6A, COL-4A, FN-1, and ACAT-2 transcripts were also found to be lowered in regard with EMS untreated livers following MSI-1436 application (*p* < 0.001).

Given the critical implication of PTP1B overexpression in liver injury and abnormal wound healing, interrelated downstream molecular factors have been furthermore analyzed.

A strong expression of PTP1B-associated fibrogenesis mediators including JAK1, JAK2, and STAT-3 (Figure 3(d)) that have been all demonstrated to promote liver fibrosis when activated through increased PTP1B activity, as well as the multicomponent nicotinamide adenine dinucleotide phosphate (NADPH) oxidase (NOX) enzyme complexes NOX-1 and NOX-4, which are strongly associated with HSC activation has been detected in EMS untreated liver explants by opposition to the healthy control group (*p* < 0.001). Notably, the inhibition of PTP1B activity using its specific competitive inhibitor trodusquemine occasioned a sizeable suppression of the aforementioned PTP1B-associated fibrosis targets as against untreated liver explants derived from EMS horses (*p* < 0.001). Therewith, the inhibitor was efficient in downregulating the relative expression of the same transcripts below the baseline when compared to healthy livers (*p* < 0.001), evoking a potential hindering effect on transcriptional events underlying liver fibrosis.

### 3.4. MSI-1436 Modulates Proteolytic Extracellular Matrix Remodeling through Matrix Metalloproteinases Regulation in EMS Liver Explants

**Figure 4:**
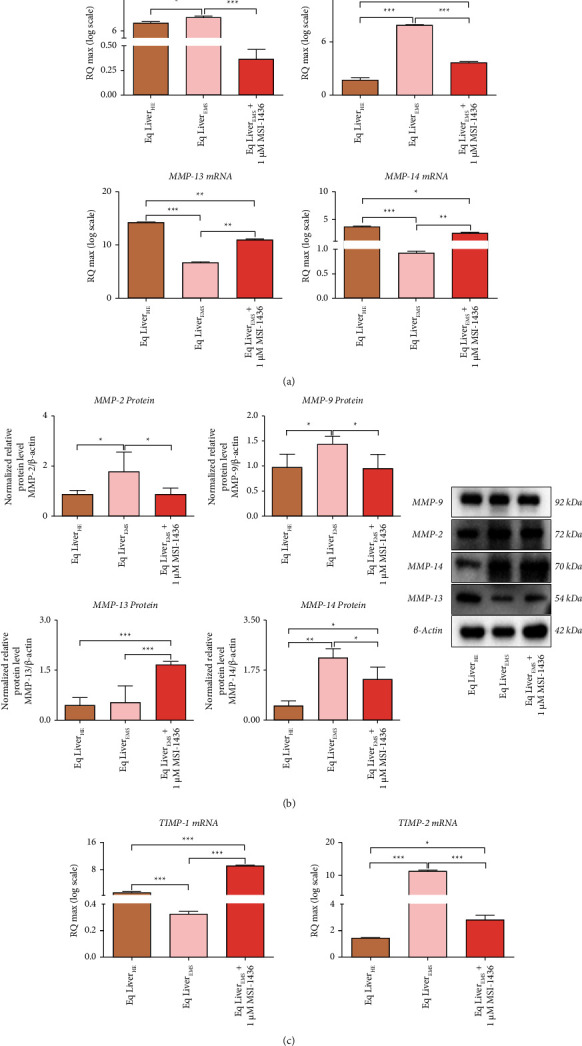
MSI-1436 regulates proteolytic ECM remodeling in EMS livers. (a) Relative gene expression of master metalloproteinases. (b) Relative protein expression of ECM-associated MMPs. Results were normalized to the expression of endogenous *β*-actin control. (c) Expression of metallopeptidase inhibitors 1 and 2. Representative data from three independent experiments are shown ± SD (*n* = 3). An asterisk (^*∗*^) indicates a comparison among healthy, EMS, and EMS-treated groups. ^*∗*^*p* < 0.05, ^*∗∗*^*p* < 0.01, and ^*∗∗∗*^*p* < 0.001. Eq Liver_HE_: liver explants derived from healthy horses; Eq Liver_EMS_: untreated liver explants derived from EMS horses. Eq Liver_EMS_ + 1 *µ*M MSI-1436: liver explants derived from EMS horses and treated with 1 *µ*M of MSI-1436 inhibitor.

Abnormal ECM remodeling represents one of the principal key events underlying progressive liver fibrosis leading to altered and aberrant deposition of extracellular proteins, proteoglycans, and carbohydrates within the liver. Thereby, the impact of PTP1B inhibition on the expression of major ECM dynamics-related drivers has been screened. EMS-affected liver tissue showed exhaustive alteration in TIMPs/MMPs expression balance (Figure 4). In point of fact, mRNA and protein levels of MMP-2 and MMP-9 were strongly elevated in the EMS group when compared to the healthy control group (*p* < 0.05; *p* < 0.001), while MMP-13 appeared to be critically downregulated at the mRNA level with no significant changes of its protein levels (Figures 4(a) and 4(b)). Interestingly, expression of MMP-14 was markedly downregulated at the mRNA level and oppositely increased at the protein level as shown in Figure 4.

Similarly, the expression level of TIMP-1 was found to be significantly lowered over an upregulation of TIMP-2, known for its stimulatory effect toward MMP-2 activation in the EMS liver (Figure 4).

Treatment of EMS liver explants with MSI-1436 resulted in an appreciable regulation of ECM proteolysis-associated effectors. The PTP1B inhibitor suppressed the increased MMP-2 and -9 expression at both mRNA and protein levels and significantly restored the expression of MMP-13 (*p* < 0.01; *p* < 0.001) when compared to the EMS untreated group and further increased its expression over the healthy group (Figures 4(a) and 4(b)). Surprisingly, while MSI-1436 improved the expression of MMP-14 transcript, a moderate reduction of its protein level was also observed in comparison with the EMS untreated group, suggesting the initiation of a transcriptional/posttranslational regulation of the proteolytic enzyme. Likewise, the application of PTP1B inhibitor potently restored the expression of TIMP-1 proteolysis inhibitor, while abolishing the increased expression of TIPM-2, which may potentially explain the observed MMP-2 lowering (Figure 4(c)) and, overall, the improvement of proper ECM remodeling machinery.

### 3.5. Inhibition of PTP1B Ameliorates Glucose Transporter 2 Stability in EMS Liver Tissue

**Figure 5:**
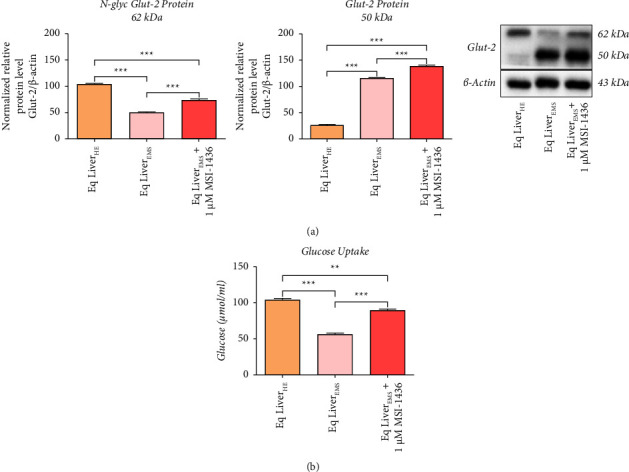
MSI-1436 ameliorates liver glucose absorption and Glut-2 availability under EMS condition. (a) Western blot analysis of the liver Glut-2 protein level. Results were normalized to the expression of endogenous *β*-actin control. (b) Histograms showing D-glucose uptake by treated and untreated liver tissue. Representative data from three independent experiments are shown ± SD (*n* = 3). An asterisk (^*∗*^) indicates a comparison among healthy, EMS, and EMS-treated groups. ^*∗*^*p* < 0.05, ^*∗∗*^*p* < 0.01, and ^*∗∗∗*^*p* < 0.001. Eq Liver_HE_: liver explants derived from healthy horses; Eq Liver_EMS_: untreated liver explants derived from EMS horses. Eq Liver_EMS_ + 1 *µ*M MSI-1436: liver explants derived from EMS horses and treated with 1 *µ*M of MSI-1436 inhibitor.

Increased PTP1B activity, persistent inflammation, and progressive fibrosis have all been reported to fundamentally contribute to insulin sensitivity loss and glucose homeostasis disruption in liver tissue. To this effect, the status of glucose transporter 2 (Glut-2) in EMS liver tissue following MSI-1436 application has been evaluated. Under EMS condition, liver tissue exhibited disrupted Glut-2 integrity, as evidenced by the decreased protein levels of deglycosylated Glut-2 over an increased in rapidly degraded nonglycosylated Glut-2 (Figure 5(a)) when compared to healthy tissue (*p* < 0.001) since N-glycosylation is a sine qua non condition for the stability of the transporter. MSI-1436 exposure resulted in an upregulation of both N-glycosylated and nonglycosylated Glut-2 protein expression when compared to both healthy and EMS controls, evoking a stimulation of the glucose absorption pathway. As a confirmation, glucose uptake analysis demonstrated that MSI-1436 treated livers absorbed significantly higher amounts of D-glucose by contrast to EMS untreated livers after 24 hrs incubation (Figure 5(b)), suggesting that PTP1B inhibition efficiently restores liver metabolic homeostasis.

## 4. Discussion

In the presented study, for the first time, we showed that MSI-1436 improves glucose uptake, modulates inflammation and inhibits fibrosis in the equine liver affected by EMS condition. MSI-1436 is a naturally occurring aminosterol that selectively inhibits protein tyrosine phosphatase 1B (PTP1B) and inactivates receptor-activated tyrosine kinases. MSI-1436 has been previously shown to protect against high-fat diet-induced obesity (DIO) in rodents model, improve insulin sensitivity mitochondrial biogenesis and dynamics, and modulate ER stress in insulin-resistant progenitor cells derived from EMS horses, and as recently showed, to promote heart injury regeneration [21, 26, 32]. Based on those discoveries, MSI-1436 is actually under clinical trials as an antidiabetic agent for humans (in accordance with ClinicalTrials.gov, 4 trials have been performed). Although its effectiveness has been demonstrated on different species, the molecular mechanisms involved in the regulation of insulin sensitivity, especially in the context of its anti-inflammatory and antifibrotic effects, have not been investigated so far. In this investigation, we have found that the *ex vivo* application of MSI-1436 in the insulin-resistant liver and peripheral blood cells isolated from EMS horses reduced proinflammatory cytokines levels, inhibited excessive liver remodeling, protected against fibrosis, and improved glucose disposal.

A growing body of evidence indicates that inflammation is becoming one of the most critical components of insulin-resistant in both humans and animals. It has been shown in multiple studies that activated immune cells through local and systemic secretion of proinflammatory factors lead to insulin resistance development [33]. Our and others' previous studies showed that EMS is strongly correlated with adipose tissue, liver, and peripheral blood mononuclear cells inflammation combined with Tregs depletion, that enhances cytokines “storm” development, leading to insulin resistance development [8, 17, 29]. In this study, we found that MSI-1436 significantly reduced the expression of IL-1*β*, IL-6, TNF-*α*, and IL-12 in the liver of EMS-affected horses. The observed effect was strongly correlated with the peripheral blood mononuclear cells' proinflammatory cytokines expression (IL-1*β*, IL-6, and IFN-*γ*) which shed promising light on the application of MSI-1436 as an anti-inflammatory agent in liver-related inflammation. Our obtained results are strongly supported by recent data from Smith and colleagues [21], who showed that MSI-1436 reduces cardiovascular inflammation during sepsis and protects against inflammation-induced gliosis in the retina. Moreover, the study, performed by Song and colleagues [34], showed that inhibition of PTP1B suppressed the overexpression levels of iNOS, COX-2, TNF-*α*, and IL-1*β* and thus protected against neuroinflammation. Interestingly, our study also demonstrated that PBMC derived from EMS-affected horses displayed reduced expression of MCP-1, a master chemoattractant cytokine. Although MCP-1 has been widely described as a proinflammatory protein excessively released during various inflammatory diseases, recent conflicting findings reported inverse connotations and showed that MCP-1 can be downregulated under excessive and prolonged inflammation. In fact, Cranford and colleagues [35] demonstrated that MCP-1 depletion leads to increased adiposity, inflated metabolic failure, aberrant macrophages migration, and exacerbated inflammation together with accelerated fibrosis and collagen type 2 thickening in a model of HFD-induced obese mice. Similarly, Kirk et al. [36] reported higher levels of macrophages infiltration along with adipose tissue expansion in MCP-1^null^ C57BL/6 mice. Taken together, these findings highlight a more complex role of MCP-1 in inflammatory processes regulation and suggest the necessity for a proper MCP-1 balance in the course of metabolic disorders such as EMS, in which MSI-1436 might be considered for the normalization of MCP-1 levels, as suggested by our obtained data that showed the positive relation between PTP1B inhibition and MCP-1 expression level. However, further investigations are needed for the elucidation of the exact mechanisms leading to both MCP-1 suppression and MSI-1436 anti-inflammatory potential in EMS horses.

The suppression and regulation of proinflammatory factors' activity might be posttranscriptionally regulated by microRNA. Thus, we showed that MSI-1436 modulates the expression of a wide plethora of miRNAs that are involved in the modulation of the liver and systemic inflammation under normal and insulin resistance conditions. In particular, we found that MSI-1436 enhanced the expression of miR-34a, which elevated expression and that has been shown to reduce the expression of IL-6 and TNF-*α* in LPS-treated RAW264.7 cells [37]. Moreover, miR-34a has been shown to be strongly related to insulin resistance development, and elevated miR-34a suppresses nicotinamide phosphoribosyl transferase (NAMPT), resulting in reduced NAD + biosynthesis in the liver of obese mice fed with high-fat diet [38]. Furthermore, we have found that inhibition of PTP1B significantly increases the expression of miR-122, in which depletion has been observed to be associated with liver hepatitis, hepatosteatosis, and the development of tumors resembling HCC [39]. Moreover, this pathological phenomenon has been shown to be closely associated with hyperactivity of oncogenic pathways and hepatic infiltration of inflammatory cells that produce protumorigenic cytokines, including IL-6 and TNF-*α*. Likewise, overexpression of miR-802 found in the EMS liver and PBMC has been reversed following MSI-1436 treatment. miR-802 has been reported to impair insulin transcription and secretion and to promote acute inflammatory response via the activation of NF-ƙB axis and downstream proinflammatory cytokines' secretion [40, 41]. Thus, it might be concluded that application of MSI-1436 in EMS horses that suffer from liver metabolism deterioration and increased inflammation could become a novel therapeutic approach.

Increased proinflammatory responses in the course of metabolic failure have also been correlated to concomitant depletion of intrinsic anti-inflammatory protective pathways [42]. In this study, MSI-1436 treatment of both the EMS liver and PBMC resulted in an enhancement of anti-inflammatory signaling. The PTP1B inhibitor increased the levels of IL-10 cytokine and more importantly activated CD4^+^CD25^+^Foxp3^+^ regulatory T cells that appeared depleted in EMS-derived PBMC. In accordance to our presented data, previous research demonstrated that PTP1B is a direct negative regulator of IL-10 and that PTP1B^(−/−)^ mice were characterized by enhanced IL-10 and IL-4R*α* expression at the mRNA and protein level [43]. Similarly, Xu et al. [44] showed that inhibition of PTP1B with punicalagin significantly improved the anti-inflammatory properties of M2 macrophages mainly via the stimulation of IL-10 and IL-4 secretion. Moreover, other PTPs have been implicated in the loss of Tregs population, as evidenced by Brownlie and collaborators [45], who reported that lack of PTPN22 increased activation and adhesion of Tregs that resealed more abundant amounts of IL-10 immunosuppressive cytokine, suggesting that PTPs are key negative regulators of immunomodulatory networks, which fine-tune the use of MSI-1436 for the management of inflammation-related malfunctions.

PTP1B has been shown as a pharmaceutical and therapeutic target for the possible treatment of obesity and type 2 diabetes or insulin resistance. However, its effect on liver fibrosis development is still elusive. Liver fibrosis has been shown to be strongly associated with type 2 diabetes, nonalcoholic steatohepatitis (NASH), or obesity development. It was shown that liver fibrosis is characterized by predominantly lobular necroinflammation, which may progress to cirrhosis and even hepatocellular carcinoma [46]. In this study, we have found that the *ex vivo* application of MSI-1436 modulates the TGF*β*-Smad2/7 axis and thus might be considered as a fibro-protective agent. PTP1B inhibition strongly reduced the expression of TGF-*β*, a master activator of hepatic stellate cells (HSC) and fibrosis initiator, and restored the suppressed expression of antifibrotic Samd7 gene. TGF-*β* is the primary profibrogenic factor initiating liver fibrosis events, through the activation of HSC trans-differentiation accompanied by the stimulation of downstream signaling pathways including Smad3/4, while inhibiting regulatory networks such as Smad2/7, which promotes fibrotic lesions progression [47]. In agreement with our observed results, Byeon et al. [48], previously found that PTP1B deficiency confers resistance to TGF-*β* profibrotic effects, while Ortiz and colleagues [49] demonstrated that loss of PTP1B reduces TGF-*β*-induced phosphorylation of Smad3 mediators, which correlated with increased levels of Smad7 and NOX axis regulation. As a matter of fact, the TGF-*β*-induced upregulation of NADPH oxidases 1 and 4 (NOX-1, NOX-4) in liver has been previously attributed to an overactivation of PTP1B [49]. In an earlier study, inhibition of NOX1/4 axis has been shown to potently mitigate and attenuate liver fibrosis and associated oxidative stress [50]. This stays in line with our obtained data, showing that PTP1B inhibition with MSI-1436 modulates NOX1/4 expression, thereby reducing fibrogenesis pathways activation.

We showed that elevated SMAD-7 expression is strongly associated with the anti-inflammatory activity of MSI-1436 and in consequence leads to reduced expression of collagen genes. It was shown that chronic inflammation can trigger an excessive accumulation of ECM components, especially collagen, the major fibrous proteins in the ECM. Prolonged inflammation that was shown in EMS horses contributes to the liver architecture remodeling by abundant accumulation of COL1A1 and COL1A2 [51]. Both collagen type turnovers are critically involved in ECM remodeling and are crucial for fibrosis development. Moreover, both COL1A1 and COL1A2 are regulated by a plethora of metalloproteinases (MMPs) that are involved in keeping the balance between collagen accumulation and degradation [52]. Apart from collagenases and gelatinases, MMP-2 and -9 have been shown to be a potential target for the treatment of liver fibrosis. MMP-2 and MMP-9 have been found to be involved in fibrosis progression as well as regression [53]. Here, we observed that MSI-1436 significantly reduces the expression of both MMP-2 and MMP-9. Moreover, PTP1B inhibition improved the expression of MMP-13 might explain the anti-inflammatory effect of MSI-1436 since macrophage MMP-13 proteinase is upregulated in response to the T helper 2-derived anti-inflammatory cytokines IL-4 and IL-13 [54]. Interestingly, we have found that the reduction of MMPs was accompanied by increased expression of tissue metalloproteinases inhibitor 1 (TIMP-1). TIMP-1 was the first discovered tissue inhibitor in the liver that inhibits collagenases and MMP-9.

Previous reports indicated that TIMP-1 suppression maybe associated with increased fibrillar collagen deposition and extensively fragmented elastic lamellae accumulation; in addition, TIMP-1 deficiency was also correlated to increased tissue inflammation evoking the importance of TIMP-1 in restricting extracellular matrix degradation and abnormal tissue scarring under pathological conditions [55]. PTP1B overexpression and/or overactivation has been previously reported to critically affect and downregulate the expression of both TIMP-1 and TIMP-2 in liver tissue and HSC [56, 57], which correlate with our observed data, in which EMS-affected livers displayed suppressed TIMP-1 gene expression.

Profound inflammation and progressive fibrosis form together a vicious circle in which persistent release of proinflammatory cytokines, macrophage infiltration, NF-ƙB/TGF-*β* activation, and pathologic tissue turnover lead to liver function collapse, metabolic imbalance, and insulin resistance [58]. Glucose transporter 2 is the major glucose transporter expressed by liver tissue, which is responsible for glucose sensing and output, and most importantly for the glucose-stimulated insulin secretion (GSIS) [59]. Lowered levels of Glu-2 have been previously associated with increased inflammation and reduced insulin sensitivity in an experimental model of diabetes [60]. Interestingly, it has been found in the present study that under EMS, Glut-2 protein undergoes disrupted posttranslational modifications. Indeed, EMS livers were characterized by lowered glycosylated Glut-2 in favour of deglycosylated Glut-2, which has been further ameliorated following PTP1B inhibition, where glycosylation of Glut-2 was increased. In agreement, Glut-2 loss has been observed in high-fat diet or glucocorticoid-induced diabetes in mice and rat models [61]. Therewith, the reduced Glut-2 availability has been suggested to be directly associated with an alteration in its extracellular loop N-linked glycosylation, which has been proved to be essential for its proper anchoring to the plasma membrane and its stability [62]. Indeed, Ohtsubo and colleagues [63] found that deficiency in N-acetylglucosaminyltransferase or posttranslational modification with glycan-ligand mimetics strongly lower the Glut-2 cell-surface half-life and subsequently lead to premature endocytosis with redistribution into endosomes and lysosomes for rapid degradation, which has been attributed to prolonged hyperglycaemia. These data together with our obtained results suggest that MSI-1436 may represent a suitable alternative for promoting cell-surface expression of the Glut-2 glycoprotein and restoring liver metabolism homeostasis.

## 5. Conclusion

MSI-1436 is a promising antidiabetic agent that has been tested in Phase 1 and 1b clinical trials. In this study, we showed that MSI-1436 reduced *ex-vivo* liver inflammation and fibrosis by activating antifibrotic factors including TIMP-1 and thus might be considered as a future molecule for the treatment of liver-related inflammation and fibrosis in equine metabolic syndrome affected horses. Moreover, as an accepted experimental model in translational medicine, our obtained data on EMS might be extrapolated to human MetS and thus serve in scaling up the ongoing clinical trials. Although clinical trials using EMS horses are necessary for further exploring MSI-1436 clinical efficacy, it seems we are way closer to solving the insulin resistance problem in horses.

## Figures and Tables

**Table 1:** List of primers used in the study.

Gene	Primer	Sequence 5′–3′	Amplicon length (bp)	Accession no.
*IL-1β*	F:	AAACAGATGAAGTGCTCCTTCCAG	391	NM_000576.3
	R:	TGGAGAACACCACTTGTTGCTCCA		

*IL-4*	F:	CTTTGCTGCCTCCAAGAACAC	97	NM_000589.4
	R:	GCGAGTGTCCTTCTCATGGT		

*IL-6*	F:	TCCTTCTCCACAAACATGTAACAA	319	NM_001318095.2
	R:	ATTTGTGGTTGGGTCAGGGG		

*TNF-α*	F:	AGTGACAAGCCTGTAGCCCA	242	NM_000594.4
	R:	GTCTGGTAGGAGACGGCGAT		

*MCP-1*	F:	ATTGGCCAAGGAGATCTGTG	167	NM_001081931.2
	R:	ATATCAGGGGGCATTTAGGG		

*CRP*	F:	TCATAGCCTTCACCGTGTGC	253	XM_023640915.1
	R:	ATGGGCTTCCCATCTACCCA		

*IFN-γ*	F:	AGCTGTGTGCGATTTTGGGT	115	NM_001081949.1
	R:	CCACCATCCCCTACATCTGG		

*IL-10*	F:	TGCTATGTTACCTGGTCTTCCTGG	461	NM_001082490.1
	R:	ACTCATGGCTTTGTAGACACC		

*TGF-β*	F:	ATTCCTGGCGCTACCTCAGT	197	NM_001081849.1
	R:	GCTGGAACTGAACCCGTTGAT		

*ACVR2A*	F:	TCTGGAAAGCCCAGTTGCTT	157	NM_001242549.2
	R:	CTGGTGCCTCGTTTTTCTGC		

*SMAD-2*	F:	AGGGTGGGGAGCAGAATACC	89	XM_023647769.1
	R:	CCAACCACTGTAGGGGTCCA		

*SMAD-7*	F:	CCCATCACCTTAGCCGACTC	164	XM_023647786.1
	R:	TTGGGAATCTGAAAGCCCCC		

*COL4A*	F:	CAGGTGAGATACTCGGCCAC	185	XM_023621826.1
	R:	CATTTGCCCCTTTTCACCCG		

*COL6A*	F:	ACAAGGGTGAGAAGGGCAAG	160	XM_001488351.5
	R:	CCTTTCAGTCCAAACGCACC		

*NOX-1*	F:	ACCACGGGATCTCAGGTTTG	166	XM_001914728.5
	R:	AAATTCAGGCAGCGAGCAGA		

*NOX-4*	F:	TGGCTCTCCTTGAATGTCCT	107	XM_001489131.5
	R:	CTTAGGCACAATCCTAGCCCC		

*JAK-1*	F:	CTGCTTTTTGGGGGACATTGG	123	XM_023641948.1
	R:	CTCCTGGTTCACCTCCGTCT		

*JAK-2*	F:	AGGGACACAAACAGGGCGT	144	XM_001492663.6
	R:	TGTTCCTCTTCCTCTTAGCCC		

*STAT-3*	F:	TGCCTGTGTTGGGTAGATGG	192	NM_001309512.1
	R:	CATCGGGAAGCTGTCACTGT		

*STAT-5*	F:	AGATGCTGGCCGAGGTCAAC	212	XM_005597383.3
	R:	AGACTTGGCCTGCTGCTCAC		

*IL-12A*	F:	GCCATCCTGGTCCTCCTAAAC	113	NM_001082511.2
	R:	AGCAGGTTTTGGGAGTGGTT		

*ACAT-2*	F:	TAGACTGAACGACGGGCTCA	181	XM_001503035.6
	R:	CACACATCTTGGCTGGAGCA		

*TIMP-1*	F:	ACTTCCACAGGTCGGAGAAC	199	XM_023633181.1
	R:	TTGCAGGGGATGGATGAACA		

*TIMP-2*	F:	TCCCTTTCGAGGGCAGACTA	170	XM_023651899.1
	R:	GGGGGTGGGGTGTTCTATTG		

*FN-1*	F:	CACACCCCAATCTTCACGGA	113	XM_023642286.1
	R:	GCCAGGAAGCTGAATACCGT		

*MMP-2*	F:	TCCCACTTTGATGACGACGA	182	XM_023637007.1
	R:	AAGTTGTAGGTGGTGGAGCA		

*MMP-9*	F:	TCGTCATCCAGTTTGGCGTT	145	NM_001111302.1
	R:	TTGCCCAGAGACCACAACTC		

*MMP-13*	F:	CACCTACACTGGCAAAAGCC	104	NM_001081804.1
	R:	TGGGATGTTTAGGGTTCGGG		

*MMP-14*	F:	CCTATGCCTACATCCGCGAG	167	XM_023621963.1
	R:	GGCAGAGTCAAAGTGGGTGT		

*PTP1B*	F:	TGGAAGGAGCTCTCCCATGA	334	XM_023626744.1
	R:	GGAAGGGCTTCCAGTGAGTC		

*VEGF*	F:	CCCACTGCGGAGTTCAACAT	221	XM_023624005.1
	R:	TTTCTCCGCTCTGAGCAAGG		

*GAPDH*	F:	GTCAGTGGTGGACCTGACCT	256	NM_001357943.2
	R:	CACCACCCTGTTGCTGTAGC		

*IL-1β*: interleukin 1 beta; *IL-4*: interleukin 4; *IL-6*: interleukin 6; *TNF-Α*: tumor necrosis factor alpha; *MCP-1*: chemokine (C-C Motif) ligand 2 (CCL2); *CRP*: C-reactive protein; *IFN-Γ*: interferon-gamma; *IL-10*: interleukin 10; *TGF-Β*: transforming growth factor beta 1; *ACVR2A*: activin A receptor type 2A; *SMAD-2*: SMAD family member 2; *SMAD-7*: SMAD family member 7; *COL4A*: collagen type IV alpha 1 chain; *COL6A*: collagen type VI alpha 1 chain; *NOX-1*: NADPH oxidase 1; *NOX-4*: NADPH oxidase 4; *JAK-1*: janus kinase 1; *JAK-2*: janus kinase 1; *STAT-3*: signal transducer and activator of transcription 3; *STAT-5*: signal transducer and activator of transcription 5; *IL-12A*: interleukin 12A; *ACAT-2*: actin, alpha 2, smooth muscle, aorta; *TIMP-1*: TIMP metallopeptidase inhibitor 1; *TIMP-2*: TIMP metallopeptidase inhibitor 2; *FN-1*: fibronectin 1; *MMP-2*: matrix metallopeptidase 2; *MMP-9*: matrix metallopeptidase 9; *MMP-13*: matrix metallopeptidase 13; *MMP-14*: matrix metallopeptidase 1; *PTPT1B*: protein tyrosine phosphatase, non-receptor type 1; *VEGF*: vascular endothelial growth factor; *miR-34*: hsa-miR-34; *GADPH*: glyceraldehyde-3-phosphate dehydrogenase.

**Table 2:** List of miRNAs used in the study.

Gene	Sequence 5′–3′	Accession no.
*hsa-mir-103a*	AGCTTCTTTACAGTGCTGCCTTG	MI0000109
*hsa-mir-155*	TTAATGCTAATCGTGATAGGGGTT	MIPF0000157
*hsa-mir-34a*	TGGCAGTGTCTTAGCTGGTTGT	MIPF0000039
*mmu-mir-211*	TTCCCTTTGTCATCCTTTGCCT	MIPF0000042
*mmu-mir-7666*	GTAGCCCGGGCGGTGCTTCCCC	MI0025006
*mmu-mir-8109*	GCGCCGCGTGCCGGCCGCGGG	MI0026041
*eca-mir-802*	CAGTAACAAAGATTCATCCTTGT	MI0012928
*hsa-mir-122*	TGGAGTGTGACAATGGTGTTTG	MI0000442
*hsa-miR-15b*	TAGCAGCACATCATGGTTTACA	MI0000438

**Table 3:** List of antibodies used in the study.

Antibodies	Concentrations	CAT numbers	Company
*β-Actin*	1 : 1000	a5441	Sigma
*Il-4*	1 : 1000	AF5142	Affinity biosciences
*Il-6*	1 : 1000	ab6672	Abcam
*IL-10*	1 : 1000	DF6894	Affinity biosciences
*MCP-1*	1 : 1000	AF5330	Affinity biosciences
*MMP-2*	1 : 1000	AF5330	Affinity biosciences
*MMP-9*	1 : 1000	HPA001238	Sigma
*MMP-13*	1 : 1000	AF5355	Affinity biosciences
*MMP-14*	1 : 1000	AF0212	Affinity biosciences
*Glut-2*	1 : 1000	orb10726	Biorbyt

*Β-Actin*: beta-actin; *IL-4*: interleukin 4; *IL-6*: interleukin 6; *IL-10*: interleukin 10; *MCP-1*: chemokine (C-C Motif) ligand 2 (CCL2); *MMP-2*: matrix metallopeptidase 2; *MMP-9*: matrix metallopeptidase 9; *MMP-13*: matrix metallopeptidase 13; *MMP-14*: matrix metallopeptidase 14; *Glut-2*: glucose transporter 2.

## Data Availability

The data that support the findings of this study are available from the corresponding author upon reasonable request.
